# Supplementary material for: The impact of human activities on Australian wildlife
Source: PLoS One. 2019 Jan 23;14(1):e0206958. doi: 10.1371/journal.pone.0206958 (PMC6344025; doi:10.1371/journal.pone.0206958)
Supplement: S1 File — (PDF) [file pone.0206958.s001.pdf]

Supporting File 1: List of species and multi-species groups (sorted into animal groups) studied between 2006 and 2017.

AVIANS

- Australian Brush Turkey *Alectura lathami*
- Australian Magpie *Gymnorhina tibicen*
- Australian Pelican *Pelecanus conspicillatus*
- Fig Bird *Sphecotheres viridis*
- Laughing Kookaburra *Dacelo novaeguineae*
- Lorikeets
  - Rainbow Lorikeet *Trichoglossus haematodes*
  - Scaly-breasted Lorikeet *Trichoglossus chlorolepidotus*
- Native Ducks
  - Pacific Black Duck *Anas superciliosa*
  - Wood Duck *Aix sponsa*
  - Whistling Ducks *Dendrocygna spp.*
- Noisy Miner *Manorina melanocephala*
- Raptors
  - Wedge-tailed Eagle *Aquila audax*
  - Whistling Kite *Haliastur sphenurus*
  - Osprey *Pandion haliaetus*
  - Brown Goshawk *Accipiter fasciatus*
  - Peregrine Falcon *Falco peregrinus*
  - Brahminy Kite *Haliastur indus*
  - Collared Sparrow Hawk *Accipter cirrhocephalus*
  - Nankeen Kestrel *Falco cenchroides*
  - Black-shouldered Kite *Elanus axillaris*
  - Australian Hobby *Falco longipennis*
  - Grey Goshawk *Accipter novaehollandiae*
  - Brown Falcon *Falco berigora*
  - Little Eagle *Hieraaetus morphnoides*
  - Spotted Harrier *Circus assimilis*
  - Swamp Harrier *Circus approximans*
  - Square-Tailed Kite *Lophoictinia isura*
  - Pacific Baza *Aviceda subcristata*
- Tawny Frogmouth *Podargus strigoides*

REPTILES

- Bearded Dragon *Pogona barbata*
- Blue-tongued Skink *Tiliqua scincoides*
- Carpet Python *Morelia spilota*
- Eastern Water Dragon *Intellagama lesueurii*
- Freshwater Turtles
  - Broad-shelled Turtle *Chelodina expansa*
  - Saw-shelled Turtle *Myuchelys latisternum*
  - Eastern Long-necked Turtle *Chelodina longicollis*
  - Krefft's turtle *Emydura macquarii krefftii*
  - Brisbane River turtle *Emydura macquarii signata*
  - Snake-Neck Macquarie turtle *Emydura macquarii macquarii*
  - Short Necked turtle *Emydura macquarii*
  - Mary River turtle (Endangered) *Elusor macrurus*
- Green Tree Snake *Dendrelaphis punctulata*
- Lace Monitor *Varanus varius*
- Venomous Snakes

- Brown Tree Snake — *Boiga irregularis*
- Eastern Brown Snake — *Pseudonaja textilis*
- Red-bellied Black Snake — *Pseudechis porphyriacus*
- Rough Scaled Snake — *Tropidechis carinatus*
- Tiger Snake — *Notechis scutatus*
- Yellow-faced Whip Snake — *Demansia psammophis*

AMPHIBIANS

- Tree frogs
  - Green Tree Frog — *Litoria caerulea*
  - Graceful Tree Frog — *Litoria gracilenta*

MARSUPIAL MAMMALS

- Bandicoots
  - Long-nosed Bandicoot — *Perameles nasuta*
  - Northern Brown Bandicoot — *Isoodon macrourus*
- Eastern Grey Kangaroo — *Macropus giganteus*
- Feathertail Glider — *Acrobates pygmaeus*
- Koala — *Phascolarctos cinereus*
- Large Gliders
  - Greater Glider — *Petauroides volans*
  - Squirrel Glider — *Petaurus norfolcensis*
  - Sugar Glider — *Petaurus breviceps*
- Marsupial Dasyurid
  - Yellow-footed Antechinus — *Antechinus flavipes*
  - Brown Antechinus — *Antechinus stuartii*
- Possums
  - Common Brushtail possum — *Trichosurus vulpecular*
  - Common Ringtail possum — *Pseudocheirus peregrinus*
  - Short-eared brushtail possum — *Trichosurus caninus*
- Small Macropods
  - Agile Wallaby — *Macropus agilis*
  - Black Striped Wallaby — *Macropus dorsalis*
  - Red-necked Wallaby — *Macropus rufogriseus*
  - Swamp Wallaby — *Wallabia bicolour*
  - Whiptail Wallaby — *Macropus parryi*
  - Red-legged Pademelon — *Thylogale stigmatica*
  - Red-necked Pademelon — *Thylogale thetis*

EUTHERIAN MAMMALS

- Echidna — *Tachyglossus aculeatus*
- Flying Foxes
  - Black Flying Fox — *Pteropus alecto*
  - Grey flying Fox — *Pteropus poliocephalus*
- Microbats
  - Eastern Broad-nosed Bat — *Scotorepens orion*
  - Gould’s Long-eared Bat — *Nyctophilus gouldi*
  - Little Broad-nosed Bat — *Scotorepens greyii*
  - Beccari’s Free-tailed Bat — *Mormopterus beccarii*
  - Eastern Free-tailed Bat — *Mormopterus ridei*
  - Eastern Long-eared Bat — *Nyctophilus bifax*
  - Eastern Forest Bat — *Vespadelus pumilus*
  - Gould’s Wattled Bat — *Chalinolobus gouldii*
  - Southern Myotis — *Myotis macropus*
