# Supplementary material for: The impact of human activities on Australian wildlife
Source: PLoS One. 2019 Jan 23;14(1):e0206958. doi: 10.1371/journal.pone.0206958 (PMC6344025; doi:10.1371/journal.pone.0206958)
Supplement: S2 File — (PDF) [file pone.0206958.s002.pdf]

Supporting File 2: List of causes for admission studied between 2006 and 2017

- Abnormal animal location
- Boat Strike
- Cat Attack
- Dog Attack
- Drowning
- Electrocuted
- Entanglements
    - Netting entanglements
    - Fencing entanglements
    - Fishing line entanglements
    - Other entanglements
- Fell out of tree
- Fire
    - Bush fire
    - Other fire
- Fishing tackle ingestion
- Hit by Car (HBC)
- Hit window
- Mower strike
- Malicious injury/ poisoning
- Natural predation
- Oiling
- Orphaned/Dependent Young
- Overt signs of disease
- Tree felling
