# Supplementary figures and images for: The impact of human activities on Australian wildlife
Source: PLoS One. 2019 Jan 23;14(1):e0206958. doi: 10.1371/journal.pone.0206958 (PMC6344025; doi:10.1371/journal.pone.0206958)

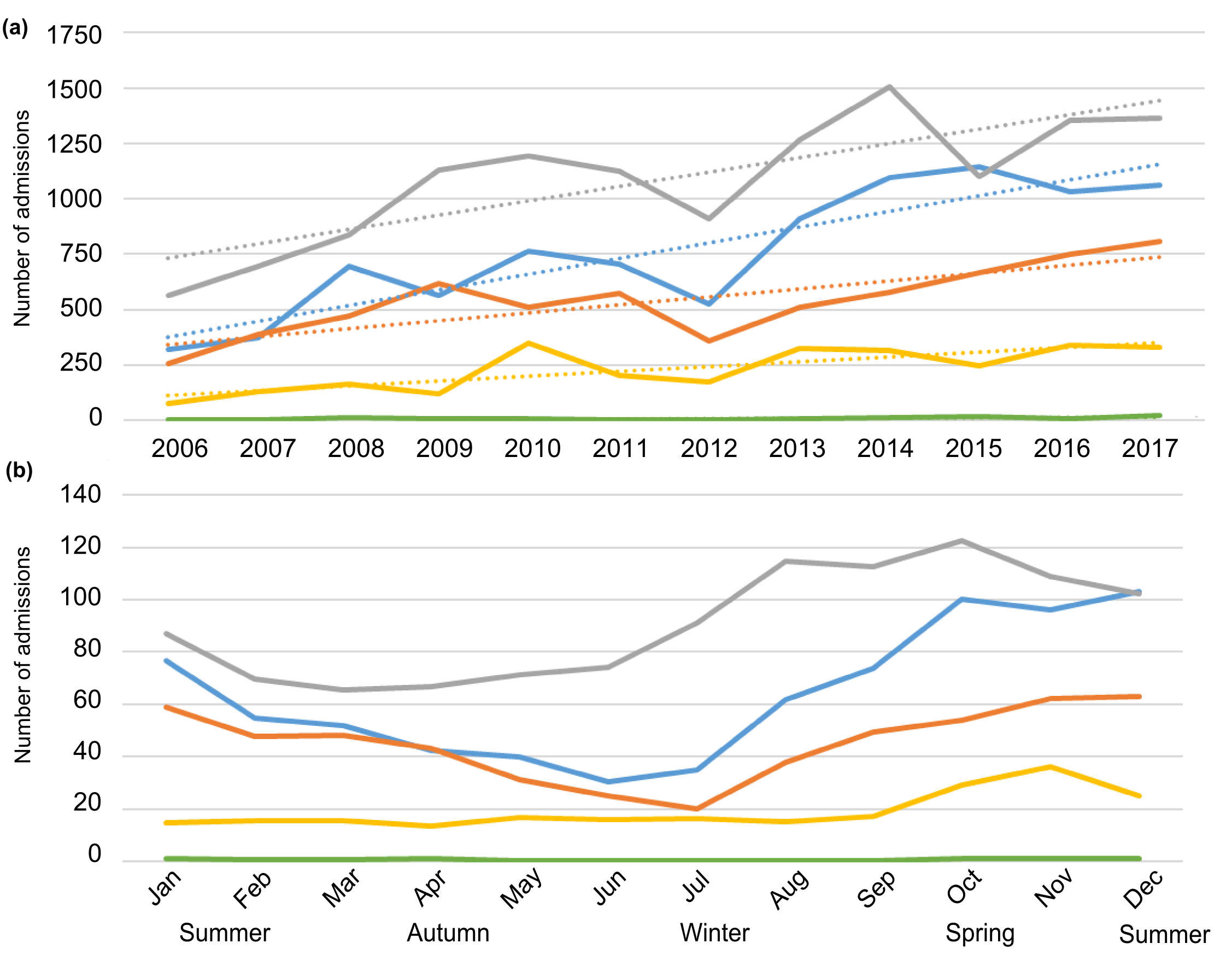

Supplement: S1 Fig — Total annual (a) and average (b) admissions per animal group. Taxa are coloured based on higher classifications; see legend. (PNG) [file pone.0206958.s008.png]

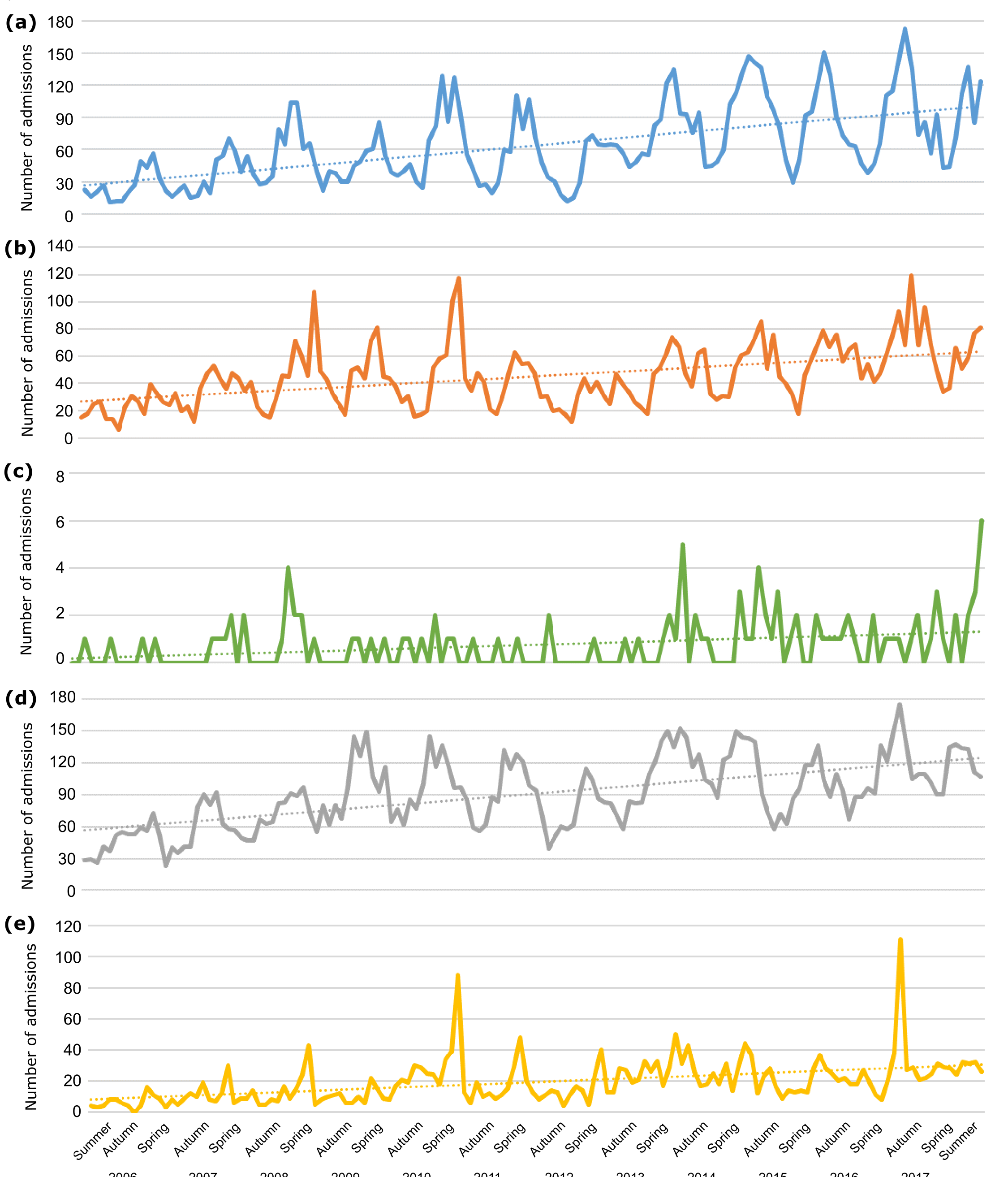

Supplement: S2 Fig — (a) avians; (b) reptiles; (c) amphibians; (d) marsupial mammals; (e) eutherian mammals. Trend lines are included to highlight the overall increase in admissions over the study period. Note the different Y axis ranges. (PNG) [file pone.0206958.s009.png]

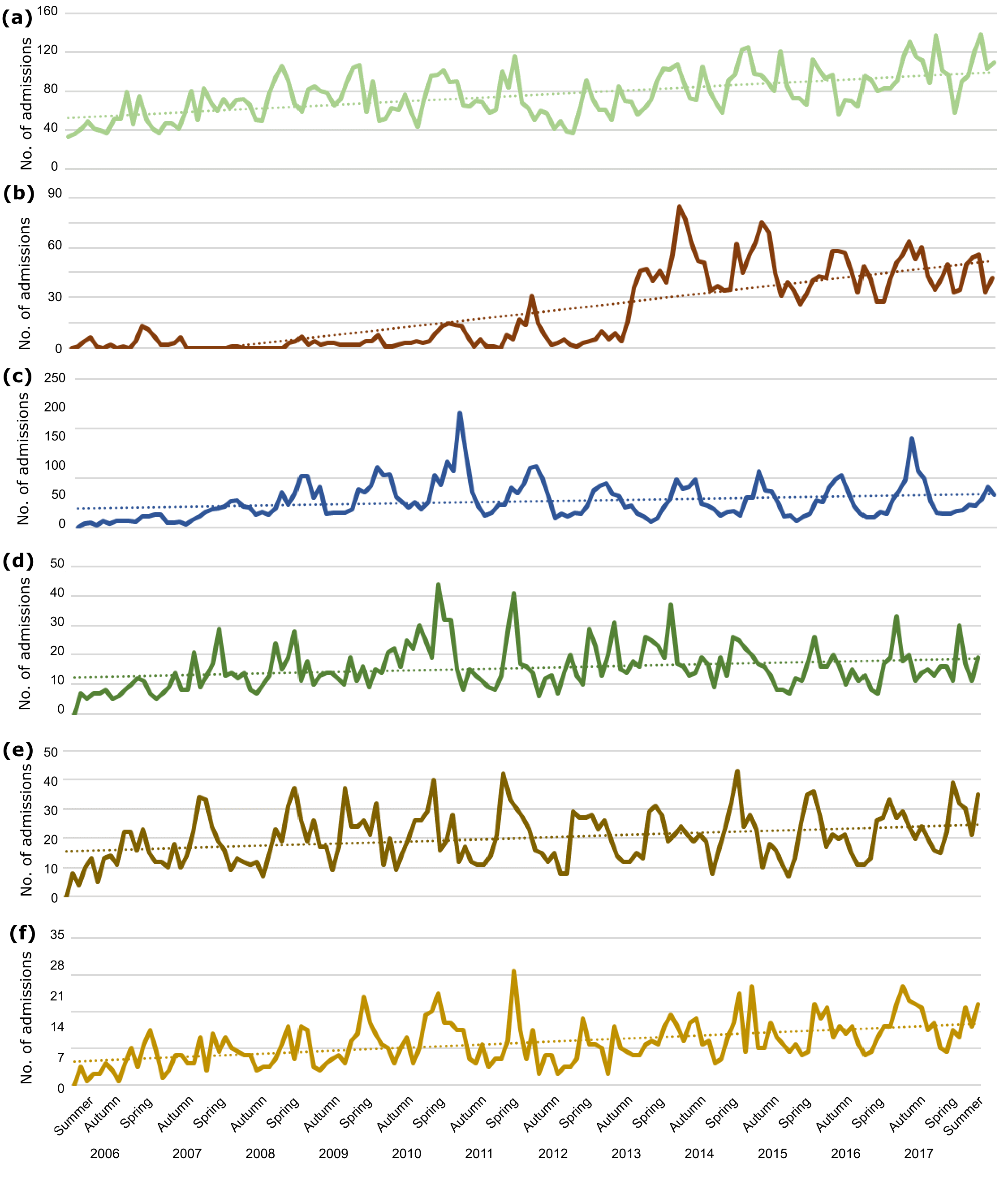

Supplement: S3 Fig — (a) hit by car; (b) overt signs of disease; (c) orphaned/dependent young; (d) entanglements; (e) dog attacks; (f) cat attacks. Trend lines are included to highlight the overall increase in admissions over the study period. Note the different Y axis ranges. (PNG) [file pone.0206958.s010.png]

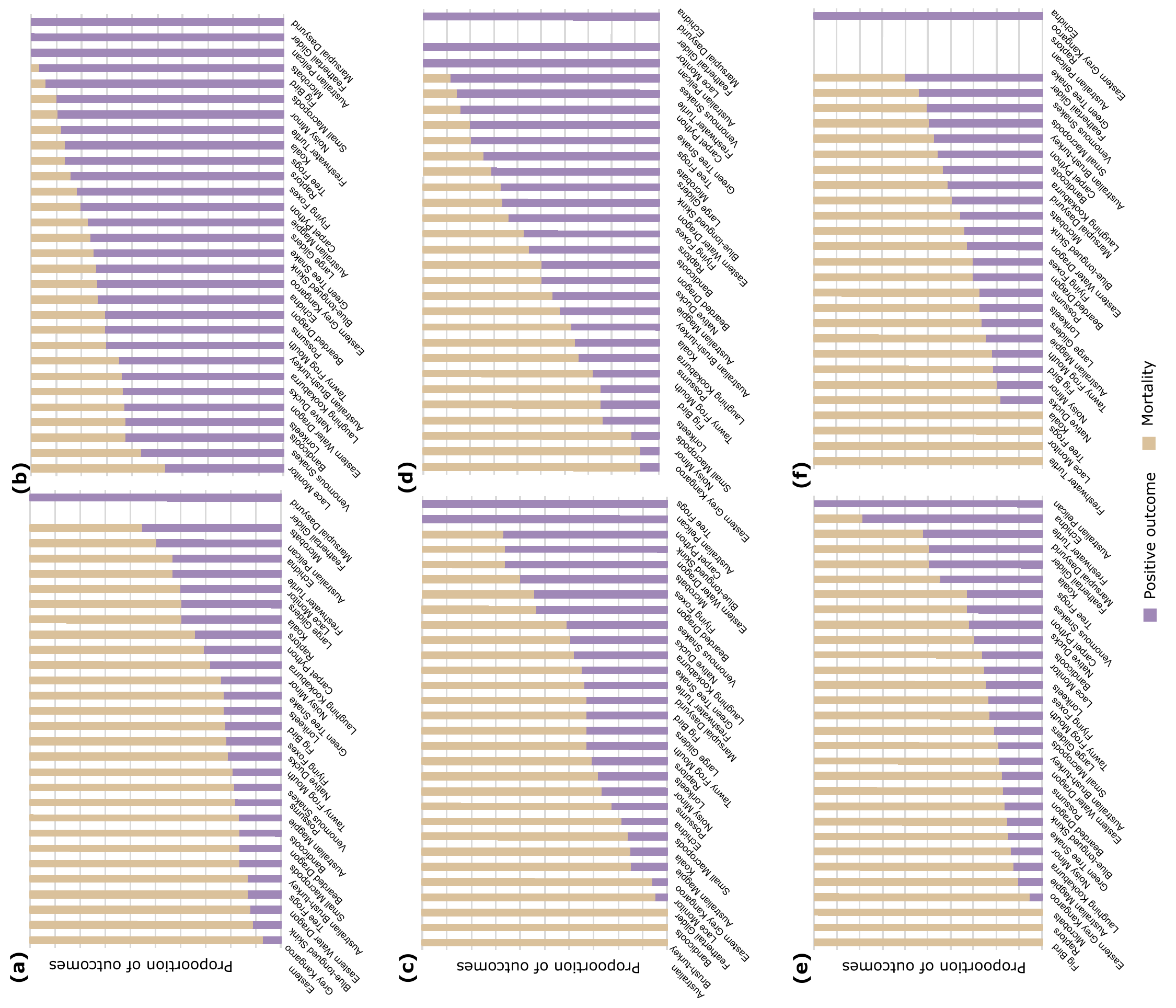

Supplement: S4 Fig — (a) hit by car; (b) overt signs of disease; (c) orphaned/dependent young; (d) entanglements; (e) dog attacks; (f) cat attacks. The mean per animal group is shown. Taxa are coloured based on higher classifications; see legend. Note the different Y axis ranges. (PNG) [file pone.0206958.s011.png]

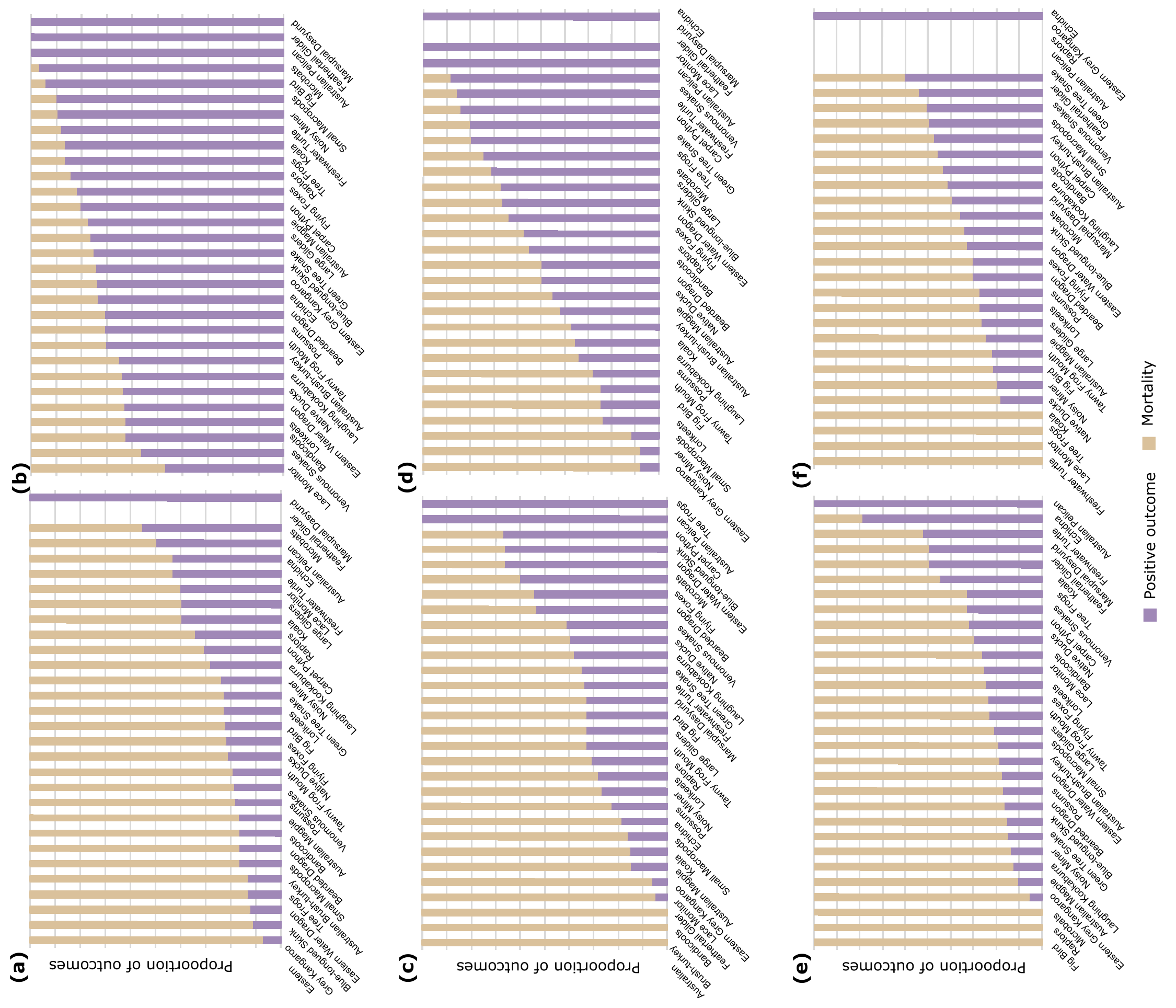

Supplement: S5 Fig — Values depicted are the proportions of total admissions for each species or multi-species group, for each CFA: (a) hit by car; (b) overt signs of disease; (c) orphaned/dependent young; (d) entanglements; (e) dog attacks; (f) cat attacks. Taxa are ordered per mortality rate (beige bars); note the different order for graphs (a) to (f). (PNG) [file pone.0206958.s012.png]
